# Outcome trajectories in a county mental health clinic before and after telemental health: a retrospective COVID-19 cohort study

**DOI:** 10.3389/fpsyg.2023.1095217

**Published:** 2023-05-16

**Authors:** Bethany A. Harris, Syed Aajmain, Adela Scharff, James F. Boswell

**Affiliations:** Department of Psychology, University at Albany, State University of New York, Albany, NY, United States

**Keywords:** telemental health, community mental health center, outcome, COVID-19, patient characteristics

## Abstract

**Background/objectives:**

Telemental health (TMH) care has received increased attention, most recently due to the COVID-19 pandemic. Many treatment settings and clinicians were forced to rapidly shift to TMH modalities, including clinicians with limited exposure to and possibly negative attitudes toward alternative treatment delivery formats. With the shift to new modalities, effectiveness research is necessary to understand if patients are receiving the same quality of care as before the pandemic and their receipt of mostly in person services. This study compared the naturalistic treatment outcome trajectories for a cohort of patients who received in-person services prior to the pandemic and a distinct cohort of patients who received TMH services after the onset of the pandemic, in a community mental health setting with limited exposure to TMH prior to the COVID-19 pandemic.

**Materials and methods:**

We adopted a retrospective cohort design to examine treatment modality as a between-group moderator of symptom change trajectory on the self-report Patient Health Questionnaire (PHQ-9) in a sample of *N* = 958 patients in the Northeast United States. Treatment durations differed in the naturalistic treatment setting and we examined patient-reported outcomes up to a maximum of one year.

**Results:**

Statistically significant average decreases in symptom severity were found over the course of up to one year of treatment, yet the average outcome trajectory was not significantly different between two modality cohorts (in person delivery before the pandemic versus TMH delivery after pandemic onset).

**Conclusion:**

These findings suggest that even in a setting with limited exposure to or training in TMH, the average outcome trajectory for patients who received TMH was statistically similar to the outcome trajectory for patients in an earlier cohort who received in-person services prior to the pandemic onset. Overall, the results appear to support continued use of TMH services in community treatment settings.

## Introduction

Although there is room for improvement, psychotherapy has demonstrated effectiveness in the treatment of a range of mental health and comorbid conditions in both controlled and naturalistic treatment settings (Barkham and Lambert, 2021). Most psychotherapy outcome studies have involved in-person, face-to-face intervention delivery formats (Barkham et al., 2021). However, telemental health (TMH) interventions have received significant attention in the past two decades (Lamb et al., 2019). Similar to face-to-face psychotherapy, TMH interventions appear to be generally effective (Bashshur et al., 2016; Hubley et al., 2016). When compared directly to more traditional in-person interventions, TMH interventions evidence similar outcomes (Backhaus et al., 2012; Varker et al., 2019).

Several factors have likely motivated increased attention toward the development, testing, and dissemination of TMH. One reason is the promise to mitigate mental health care access problems (Dowling and Rickwood, 2014; Olfson et al., 2019). Unmet treatment needs are especially prominent in elderly populations, those who identify as a racial-ethnic minority, low-income individuals, and those who reside in rural areas (Wang et al., 2005; Olfson et al., 2019). Despite the promise of improving access, in general as well as in specific populations, there are limitations to existing research on TMH outcomes and knowledge gaps remain.

To our knowledge, naturalistic TMH implementation has been examined most extensively in the United States (U.S.) in the context of the Veterans Administration (Offering Veterans VA Care Closer to Home, 2021). A recent cohort study of rural U.S. Veterans found that dissemination of internet-ready tablets for TMH in the context of the COVID-19 pandemic was associated with reduced suicidal behavior and emergency department visits (Gujral et al., 2022), Much of the other evidence regarding TMH effectiveness in the U.S. is derived from controlled studies involving homogenous patient samples (Schwartzman and Boswell, 2020). In addition, and not surprising, TMH efficacy research has mostly relied on clinicians with both interest and at least some degree of credentialed training in delivering interventions in TMH formats (Varker et al., 2019). In contrast to this self-selection, the COVID-19 pandemic required most mental health care systems and professionals to shift to TMH more or less overnight (Pierce et al., 2020; Perle, 2022), and TMH use peaked during the pandemic (Torous et al., 2020).

Pierce et al. (2020) conducted a survey of psychologists who *did not* use TMH prior to the COVID-19 pandemic. Among the most endorsed reasons for not using TMH prior to the pandemic were insufficient training, privacy issues, unclear reimbursement practices, efficacy concerns, and insufficient demand. Interestingly, a different survey of over 400 therapists with diverse training backgrounds indicated that most therapists reported having *some* degree of past TMH training/education (e.g., a workshop), yet less than half reported using TMH prior to the COVID-19 pandemic (Perle, 2022). Similar studies suggest that prior to COVID-19 approximately 20% of psychologists had used TMH at any frequency in their practice (Glueckauf et al., 2018). Low rates of pre-COVID-19 TMH adoption may be partly explained by some clinicians possessing negative, or at least ambivalent, attitudes toward TMH (Adler-Milstein et al., 2014; Wade et al., 2014). Some findings indicate that *patients* of color espouse concerns about the quality of TMH compared with in-person services (George et al., 2012). Recent research on potentially shifting clinician attitudes toward TMH has highlighted that clinicians perceive both advantages (e.g., improvements in access to services) and disadvantages (e.g., concerns about alliance quality) to increasing reliance on TMH (AlRasheed et al., 2022; Lipschitz et al., 2022).

Increasing our knowledge of mental health care stakeholder attitudes and experiences regarding the increased reliance on TMH is important. In addition, there is a need for more research on the *effectiveness* of TMH in routine community mental health settings. Naturalistic outcome studies in this area are lacking and even less is known about outcomes in more diverse community settings. COVID-19 has raised additional interest in understanding potential outcome differences among treatment modalities. Specifically, for many systems that were required to shift rapidly to TMH in the context of COVID-19, it is unclear if patients treated via telehealth after the onset of COVID-19 experienced similar, worse, or better outcomes than patient cohorts that were treated in-person prior to COVID-19. The current study investigated patient reported outcome trajectories in a mental health clinic in the context of their rapid shift to TMH at the start of the COVID-19 pandemic.

The current study is a continuation of a practice-research partnership between a county mental health clinic and psychotherapy researchers in the Northeast United States. The clinical context is a public supported mental health clinic that provides outpatient mental health services to under-resourced individuals in the community, many of whom suffer from a severe and persistent mental illness. Prior to the COVID-19 pandemic, psychotherapy interventions in this setting were universally delivered in-person. Within a few days of the onset of the pandemic, psychotherapy interventions became universally telehealth in modality, including both telephone and videoconferencing formats. Despite a general awareness of the emergence and reported effectiveness of TMH, stakeholders in the setting were skeptical of TMH and did not pursue targeted or rigorous training in TMH prior to COVID-19. Based on personal communications with clinic administrators, the existing concerns were consistent with published survey research (e.g., Connolly et al., 2020; Lipschitz et al., 2022). Given their rapid shift to TMH, administrators were interested in examining their own routinely collected patient reported outcomes for cohorts of patients who were seen before versus after the implementation of TMH. As part of the ongoing practice-research partnership, clinic stakeholders provided permission for researchers to use some of their routinely collected data to investigate potential differences in patient reported outcomes in the context of the pandemic prompted move to TMH.

Based on routinely collected data from this clinic, the present practice-oriented research study aimed to explore trajectories of change in the clinic’s primary repeated outcome measure (Patient Health Questionnaire, PHQ-9; Kroenke et al., 2001), with particular attention to in-person vs. TMH services (or pre- versus post-COVID-19 patient cohorts) as a between-group moderator of change trajectory. Using a retrospective cohort analytic design, we explored if group-level outcome trajectories differed as a function of treatment modality.

Given existing research on the effectiveness of TMH, we expected that the overall trajectory of change would be similarly positive between in-person pre-COVID and telehealth post-COVID onset cohorts. Notably, however, the unique features of this setting rendered this expectation tentative. Prior to COVID-19, anecdotally, attitudes toward TMH in this setting were mixed at best. In addition, exposure to TMH training was extremely limited. Finally, this urban setting serves a relatively higher proportion of economically disadvantaged individuals with severe and persistent mental illness who are less represented in the existing TMH research (Schwartzman and Boswell, 2020).

## Materials and methods

### Participants

Data were derived from the routine data collection infrastructure of an outpatient community mental health clinic (CMHC) in the Northeastern United States. This CMHC provides treatment to adult county residents with serious mental illness and substance use disorder diagnoses. This setting collects routine data from patients to monitor treatment processes and outcomes and inform quality improvement. Adult patients at the CMHC between September 2017 and August 2021, and who completed the PHQ-9 at baseline and at least one follow-up timepoint (*N* = 958) were included in the current study. Patients were excluded if they were missing any PHQ-9 score or if they had a baseline value yet no follow-up data. Additional demographic information was obtained from records kept by the facility. Demographic information is typically collected in the context of the initial intake appointment and is expected to be entered into an administrative database by the assigned clinician. Although the date of service when a PHQ-9 questionnaire was administered and a baseline PHQ-9 score was available for all patients included in the analyses, other demographic variables had significant missingness. Racial/ethnic identity was not recorded for most of the sample, and among those for whom racial/ethnic identity data were available (*n* = 206), racial/ethnic identity was coded as unknown for 18.4% of cases. Among the remaining (*n* = 168) patients with known racial/ethnic information, 60.7% were recorded as White, 32.2% as Black, 3.0% as another race or mixed races, 2.4% as Hispanic/Latinx, and 1.7% as Asian or Asian-American. Approximately half of those whose sex was available (*n* = 268) were recorded as male (55.2%) and the remainder were recorded as female. Patient age ranged from 19–77 (*n* = 263, *M* = 46.22, *SD* = 13.70).

New patients are expected to be given a clinician-assigned primary diagnosis based on the Diagnostic and Statistical Manual of Mental Disorders-5 (American Psychiatric Association, 2013). Like the demographic information in the administrative database, many patients did not have a recorded primary diagnosis. For those who had an assigned and recorded diagnosis in the database (*n* = 236), the most common primary diagnoses were in the categories of mood disorders (44.5%) or psychotic disorders (42.4%). The remainder of patients had primary diagnoses of trauma-related disorders (8.1%), anxiety disorders (3.0%), substance use disorders (1.3%), or other disorders (0.8%). Notably, a relatively small percentage of patients in the study database were assigned a *primary* diagnosis of a substance use disorder. Patients with more severe and acute substance-related problems typically receive services in a different affiliated clinic.

### Measures

This study evaluated whether treatment modality (before versus after the onset of the COVID-19 pandemic cohort/use of TMH), race/ethnicity (White vs. non-White identifying), and/or their interaction moderated cohort trajectories of symptom change during routine outpatient treatment.

### Treatment modality/cohort

Treatment modality (in-person services versus TMH) was nested within pre- versus post-COVID-19 pandemic onset, such that each case was coded 1 for pre- and 0 for post-COVID treatment. Patients who initiated and completed a course of treatment prior to 3/1/2020 were categorized and dummy-coded as pre-pandemic onset/in-person service cases (*n* = 738). Patients who began treatment after 3/1/2020 were categorized and dummy-coded as post-pandemic onset/TMH cases (*n* = 220). This time demarcation reflected the full transition to offering psychotherapy via telehealth at the CMHC.

### Outcome

The outcome variable was the PHQ-9 (Kroenke et al., 2001), which is a 9-item self-report measure of depression symptom severity widely used as a screening and outcome monitoring measure in primary care and mental health care settings (Kroenke, 2021). Items correspond to the DSM criteria for major depressive disorder. Patients rate the frequency with which they have experienced each of these symptoms during the past two weeks on a scale from 0 (“not at all”) to 3 (“nearly every day”). The PHQ-9 has good internal consistency reliability, with *alpha* between 0.80 and 0.90 (Kroenke et al., 2001; Levis et al., 2019). Originally developed as a depression screening tool, the PHQ-9 has been validated in psychiatric settings and shows good sensitivity to change among patients with diverse psychiatric disorders (Beard et al., 2016). The PHQ-9 is widely used as a general measure of mental health status (e.g., Bone et al., 2021). Recent findings show that in general mental health settings the PHQ-9 functions more as a general measure of symptoms/distress than as a disorder-specific scale, and it may be most appropriate as an outcome monitoring tool in settings where diagnoses are less precise and comorbidity is common (Katz et al., 2021).

### Procedures

New patients provided written and informed consent for the clinic to collect and use their routine clinical information for administrative review and quality assessment and improvement purposes. This study was approved as an exempt research project by a university institutional review board (IRB). Diagnoses were assigned by clinicians upon patients’ first visit to the CMHC, and patients are administered the PHQ-9 at intake and then throughout treatment. Clinicians are expected to readminister the PHQ-9 on an approximately monthly basis; however, it is up to the clinician’s discretion regarding whether an assessment will be conducted at a particular visit (e.g., may not be administered in a state of crisis). Given the naturalistic setting and the varied nature of the psychotherapies implemented, session frequencies and treatment durations vary among patients. Consequently, there was variability in PHQ-9 data collection.

Furthermore, given the level of impairment of some patients in this setting, information can be collected verbally rather than in patient-completed written form, and regardless of format, PHQ-9 total scores are entered by the clinician in the administrative database. The standard in the setting is for patients to complete measures in a self-report format in the waiting area (pre-COVID-19). However, even prior to the pandemic, clinicians were allowed to administer questionnaires or forms verbally and record responses if deemed more appropriate. When the current treatment setting moved to TMH, the PHQ-9 was administered verbally by clinicians and the scores were recorded in the database. However, it was not the case that all pre-TMH PHQ-9 administrations were more “traditional” self-report administrations. Unfortunately, the precise format of each PHQ-9 administration was not recorded, neither before nor after the move to TMH.

### Treatment

All patients received individual psychotherapy services from licensed psychotherapists through the CMHC. Patients were eligible to receive additional services including medication management, group psychotherapy, and treatment planning. Prior to COVID-19 onset, individual psychotherapy took place via in-person sessions and the clinic did not offer telepsychotherapy. In March of 2020, the clinic transitioned to TMH following public health guidance. Although details about the particular treatments delivered were not collected or available, setting staff describe the approach as largely supportive and problem-focused. In the current participant sample, all therapy providers were licensed Masters-level clinical social workers. Although information regarding a particular therapist’s theoretical orientation is/was not collected, the predominant orientation is best characterized as integrative, as staff are described as drawing from a mix of supportive, trauma-informed, solution-focused, and third-wave cognitive behavioral therapy approaches.

### Data analysis

Analyses were conducted using SPSS version 25. Multivariate normality was inspected within groups of interest. The PHQ-9 total score was the longitudinal outcome variable of interest, and all included patients had a baseline and follow-up PHQ-9 score. Given the naturalistic variability in treatment duration and PHQ-9 observations, we examined these features and observed a large range in both domains. Based on this and input from setting administrators, we applied an additional inclusion/exclusion criterion: for cases with treatment courses that went beyond one year, we excluded PHQ-9 *observations* past the one-year mark. This affected *n* = 348 cases. Notably, no *cases* were removed from the analysis; rather, we elected to remove outlying time points. This increased the consistency between the groups. As expected, cases with trimmed observations (due to a course of treatment exceeding one year) had significantly longer treatment durations than cases with untrimmed observations (*p* = 0.00). The average number of PHQ-9 observations in the post-COVID onset/TMH cohort was slightly higher (*M* = 3.18, *SD* = 2.62; range = 2–26; *Median* = 2.00; 25% = 2.00, 75% = 3.75) than the pre-COVID/in-person cohort (*M* = 3.12, *SD* = 1.77, range = 2 to 20; *Median* = 2.00 25% = 2.00, 75% = 4.00).

Given the multilevel data structure with PHQ-9 scores nested within patients, multilevel models (MLM; Raudenbush and Bryk, 2002) were used to test the primary research question. MLMs are suited for longitudinal data analysis as they are robust to the data dependency. MLMs are efficient in handling missing and unevenly spaced data by using all available data for a given participant to estimate group trends at each time point, making this a particularly suitable approach in this context. Maximum likelihood and an unstructured covariance were used as the estimation method, as well as random intercepts and slopes centered at baseline. Our primary analysis involved one multiple predictor model focused on the pre- versus post-COVID onset (in-person versus TMH cohort) predictor and moderator. Prior to testing this model, we explored the best fitting base model for time coded as the occasion of observation and centered at baseline. The difference between the linear and linear plus quadratic time models exceeded the critical value, so the non-linear time effect was retained in the model. The primary multilevel model included the main effect of treatment modality (pre- versus post-pandemic/TMH onset), linear time, quadratic time, the interaction between treatment modality and linear time, and the interaction between treatment modality and quadratic time. In addition, we tested pattern mixture models to examine if missing value pattern significantly influenced the association between treatment modality and PHQ-9 trajectories (Hedeker and Gibbons, 1997). In each case, the addition of the missing value effects did not result in significantly improved model fit. In addition, the fixed effect interaction with missing pattern was not statistically significant.

## Results

### Treatment modality cohort descriptives

We explored available demographic and clinical information in both modality cohorts. Group-level descriptives are reported in Table 1, along with inferential test results where applicable (e.g., some non-binary race/ethnicity and diagnostic categories had too few cases). The average baseline PHQ-9 scores in both cohorts were in the mild-to-moderate severity range (Kroenke et al., 2001); the pre-COVID cohort evidenced higher baseline scores. In addition, even with capping treatment duration at one year, we observed a statistically significant difference in treatment length between pre-COVID and post-COVID onset cohorts, with pre-COVID cases averaging many more days in treatment (values represent time in treatment and not number of treatment sessions). In addition, the pre-COVID onset cohort was older in age. We did not observe a difference on dichotomized racial/ethnic minority status between cohorts. Based on comparisons between cases with trimmed and untrimmed observations and the modality cohorts, we included grand mean centered baseline PHQ-9 score, age, and treatment duration as covariates in the primary model.

**Table 1:** Pre- and post-COVID onset cohort demographic information.

Variable	Pre-COVID	Post-COVID		
	(total *n* = 738)	(total *n* = 220)		
Category	*M* (SD)	*M* (SD)	*t*-test	*p*
	*n* (%)	*n* (%)	χ^2^/Fisher’s exact test	
Baseline PHQ-9	7.96 (6.89)	7.02 (6.15)	*t*(956) = −1.83	0.068
Minimum	0	0		
Maximum	27	24		
*Gender*				
Male	130 (53.9%)	18 (66.7%)	*X*^2^ (1, *n* = 268) = 1.59	0.207
Female	111 (46.1%)	9 (33.3%)		
*Minority Status*				
White	94 (61.0%)	8 (57.1%)	*X*^2^ (1, *n* = 168) = 0.08	0.775
Non-White	60 (39.0%)	6 (42.9%)		
*Diagnosis*				
Anxiety	6 (2.8%)	1 (4.2%)		NA
Mood	92 (12.5%)	13 (54.2%)		
Trauma	18 (2.4%)	1 (4.2%)		
Psychotic	93 (12.6%)	7 (29.2%)		
Substance Use	2 (0.3%)	2 (8.3%)		
Other	1 (0.1%)	0 (0.0%)		
Age (Years)	46.79 (13.73)	41.26 (12.63)	*t*(261) = −2.00	0.047
Treatment Duration (Days)	126.31 (129.97)	47.15 (78.55)	*t*(956) = −8.58	0

Means and standard deviations reported for continuous variables. *T*-values and *p*-values of independent samples *t*-tests are reported for continuous variables. Sample sizes and percentages are reported for categorical variables. *χ*^2^-values and *p*-values of Chi-squared tests and *p*-values of Fisher’s exact tests are reported for categorical variables.

### Pre-post COVID-19 onset/telemental health modality cohort model

Model results are reported in Table 2. For the primary predictors of interest, the linear time effect was statistically significant, indicating that, on average, patients experienced improvements in their symptoms over the course of treatment. However, the main effect of quadratic time was not statistically significant. The main effect of treatment modality (in-person versus TMH cohort) was not statistically significant (*p* = 0.868). In addition, the interaction effect between linear time and modality cohort was not statistically significant (*p* = 0.346), and the interaction between quadratic time and modality cohort was not statistically significant (*p* = 0.412).

**Table 2:** COVID-19 onset/treatment modality effects on cohort outcome trajectories.

Parameter	Coefficient	*df*	*t*	*p*	95% CI
Intercept	7.47	706.83	9.53	0.000	5.93, 9.00
Age	−0.41	253.48	−2.27	0.024	−0.76, −0.06
Treatment Duration	0.08	267.54	0.37	0.712	−0.33, 0.48
Baseline PHQ-9	4.06	253.24	23.65	0.000	3.73, 4.40
Time	−2.11	687.86	−2.61	0.009	−3.69, −0.52
QuadTime	0.24	714.73	1.58	0.114	−0.06, 0.54
COVID/Modality	−0.14	702.05	−0.17	0.868	−1.78, 1.49
Time*COVID/Modality	0.80	688.18	0.94	0.346	−0.87, 2.48
QuadTime*COVID/Modality	−0.13	720.40	−0.82	0.412	−0.45, 0.19

Modality, binary predictor indicating pre- versus post-COVID-19/telemental health modality cohort; PHQ, Patient Health Questionnaire; Time, Linear time effect; QuadTime, Quadratic time effect.

## Discussion

Although evidence for the effectiveness of TMH is encouraging, and controlled research often demonstrates similar outcomes between in-person and TMH interventions, previous studies have typically involved trained and motivated telehealth clinicians and homogenous patient samples. Furthermore, quantitative and qualitative research demonstrates that many patients, therapists, and administrators remain skeptical of TMH. However, attitudes toward TMH may be shifting out of necessity, in the context of the COVID-19 pandemic that forced most service providers to rapidly adjust from in-person to TMH services. Some research has examined the experiences and “lessons learned” of stakeholders, yet less has been published on patient outcomes in routine service settings in the context of COVID-prompted practice changes.

The current study explored the potential impact of the rapid shift to TMH in a CMHC setting that did not offer TMH services prior to the onset of the COVID-19 pandemic, with a comparison of average outcome trajectories between pre- and post-COVID-19 onset patient cohorts. On average, patients receiving psychotherapy in this CMHC demonstrated significant symptom improvement regardless of treatment modality. Average outcome trajectories were positive and did not systematically differ between modality cohorts in this context. The absence of a statistically significant difference is consistent with prior research demonstrating that TMH often yields similar effects to in-person mental health services (Backhaus et al., 2012; Varker et al., 2019).

Although analyses examining the effect of treatment modality did not indicate a statistically significant difference in average symptom *trajectories* between the modality cohorts up to one year in treatment, patients in the pre-COVID cohort presented with somewhat higher baseline severity on the PHQ-9 relative to the post-COVID-19 onset cohort (*d* = 0.13). This result is somewhat counterintuitive given other reports of increasing levels of anxiety and depression in the context of the pandemic (e.g., Bueno-Notivol et al., 2021). Several factors may have contributed to this observed difference, including potential differences between surveys involving broader community samples versus assessments of treatment-seeking clinical samples. Offering psychotherapy via TMH may have improved access to treatment due to the ability of TMH to lessen barriers to and increase reliability of accessing care. This may be especially true among under-resourced individuals in the community such as those served by the clinic. By having access to one’s therapist at the “push of a button,” barriers such as cost or travel are likely reduced. Without having to leave home, there may be reduced stress associated with needing to find childcare or take extra time away from work, both of which otherwise add to the hardships that may already be experienced by marginalized communities (Hilty et al., 2007; Pruitt et al., 2014).^1^ Overall, these findings provide further support for the generalizability of the effectiveness of TMH as part of routine care in CMHC settings.

### Strengths and limitations

The present study had several strengths. This study used naturalistic clinical data from the delivery of psychotherapy as part of routine care and had a large overall sample. It also had broad inclusion criteria, with diverse diagnoses and ethnicities observed in the patient sample. These factors likely enhance the ecological validity of the study and findings, supporting the generalizability of the findings to patients in similar CMHCs. Findings also have potential to inform decisions regarding services moving forward at this clinic, such as continuing or possibly expanding TMH.

However, the current study also had several limitations. First, we cannot draw conclusions about the precise nature of the interventions delivered, beyond involving psychotherapy in different modalities. Second, there was a substantial amount of missing data, particularly for patient characteristic variables. Data may be missing due to administrative error or oversight; in addition, there may have been some data loss when the setting changed electronic records systems. Third, we do not know the precise method of assessment for each case or time point when services were previously provided in-person. Fourth, this study did not involve random assignment to in-person or TMH. We were, however, able to take advantage of the clear demarcation of TMH implementation, akin to an interrupted time series. Fifth, we did not have access to therapist data, which prevented us from including therapists in our model and testing potential therapist effects. Sixth, given the naturalistic setting, there was a large range in treatment duration and assessment frequency, so we applied a cutoff to reduce some degree of heterogeneity across the sample and groups of interest. The findings are limited to what was observed through up to one year of treatment (see plot of raw scores in Online Supplementary Figure S1). Finally, it is important to note that the current study examined differences at the between group/cohort level and focused on group level-average trajectories. This masks meaningful heterogeneity in response trajectories among different groups of patients.

## Conclusion

To our knowledge, this is one of a limited number of studies on the impact of the COVID-19 pandemic and the rapid move to TMH focused on pre- versus post-COVID-19 onset outcomes in routine mental health treatment. Findings add to the growing empirical support for TMH. Results suggest that TMH is a generally effective treatment modality for providing psychotherapy to a range of patients. Future research should focus on unpacking the heterogeneity of modality effects in naturalistic samples. Assuredly some patients in each cohort declined in status over the course of treatment while others improved more substantially. In turn, there may be patients who have a similar likelihood of responding to either modality. It will be important to disentangle this variability and to identify patients for whom in person services (or telehealth) are likely to be of most benefit.

## Data availability statement

The raw data supporting the conclusions of this article will be made available by the authors upon reasonable request.

## Ethics statement

The studies involving human participants were reviewed and approved by University at Albany Institutional Review Board. Written informed consent for participation was not required for this study in accordance with the national legislation and the institutional requirements.

## Author contributions

BH assisted with data collection and management, conducted the data analysis, and assisted in all aspects of the writing process. AS and SA participated in the writing process and contributed as experts. SA also assisted with data analysis. JB facilitated data collection, sharing, developed study objectives, and participated in the writing process. All authors contributed to the article and approved the submitted version.

## Conflict of interest

The authors declare that the research was conducted in the absence of any commercial or financial relationships that could be construed as a potential conflict of interest.

## Publisher’s note

All claims expressed in this article are solely those of the authors and do not necessarily represent those of their affiliated organizations, or those of the publisher, the editors and the reviewers. Any product that may be evaluated in this article, or claim that may be made by its manufacturer, is not guaranteed or endorsed by the publisher.

## Footnotes

^1^We conducted an exploratory model that examined the effect of racial/ethnic minority status on PHQ-9 outcomes. We failed to find a significant main or interacting effect of this variable. Given the very small sample with available demographic information in the post-COVID onset group, these results must be interpreted cautiously. Please see the Online Supplement for full model results.
