# Supplementary material for: Outcome trajectories in a county mental health clinic before and after telemental health: a retrospective COVID-19 cohort study
Source: Front Psychol. 2023 May 16;14:1095217. doi: 10.3389/fpsyg.2023.1095217 (PMC10227450; doi:10.3389/fpsyg.2023.1095217)

**Online Supplement**

**Table S1**

*Binary Patient Race/Ethnicity Effects on Outcome Trajectories*

| Parameter | Coefficient | *df* | *t* | *p* | 95% CI |
| --- | --- | --- | --- | --- | --- |
| Intercept | 6.70 | 287.06 | 13.56 | .000 | 5.73, 7.68 |
| Age | -0.40 | 170.81 | -1.84 | .068 | -0.82, 0.03 |
| Treatment Duration | 0.22 | 187.73 | 0.85 | .397 | -0.29, 0.72 |
| Baseline PHQ-9 | 4.29 | 185.01 | 19.07 | .000 | 3.84, 4.73 |
| Time | -1.20 | 399.44 | -3.28 | .001 | -1.92, -0.48 |
| QuadTime | 0.09 | 451.10 | 1.13 | .258 | -0.06, 0.23 |
| Race/Ethnicity | 0.14 | 419.27 | 0.23 | .820 | -1.03, 1.30 |
| Time*Race/Ethnicity | -0.12 | 401.75 | -0.19 | .849 | -1.30, 1.07 |
| QaudTime*Race/Ethnicity | 0.05 | 435.59 | 0.39 | .696 | -0.20, 0.31 |

*Note.* Race = Binary White vs non-White identifying; PHQ = Patient Health Questionnaire

Time = Linear time effect; QuadTime = Quadratic time effect

**Figure S1**

Plots of observed Patient Health Questionnaire (PHQ-9) values in both Pre-COVID Onset (in person modality) and Post-COVID Onset (telehealth modality) cohorts. The x-axis represents the number of measurement occasions, which varied by patient. The y-axis represents PHQ-9 total severity score, with higher scores indicating greater clinical severity.


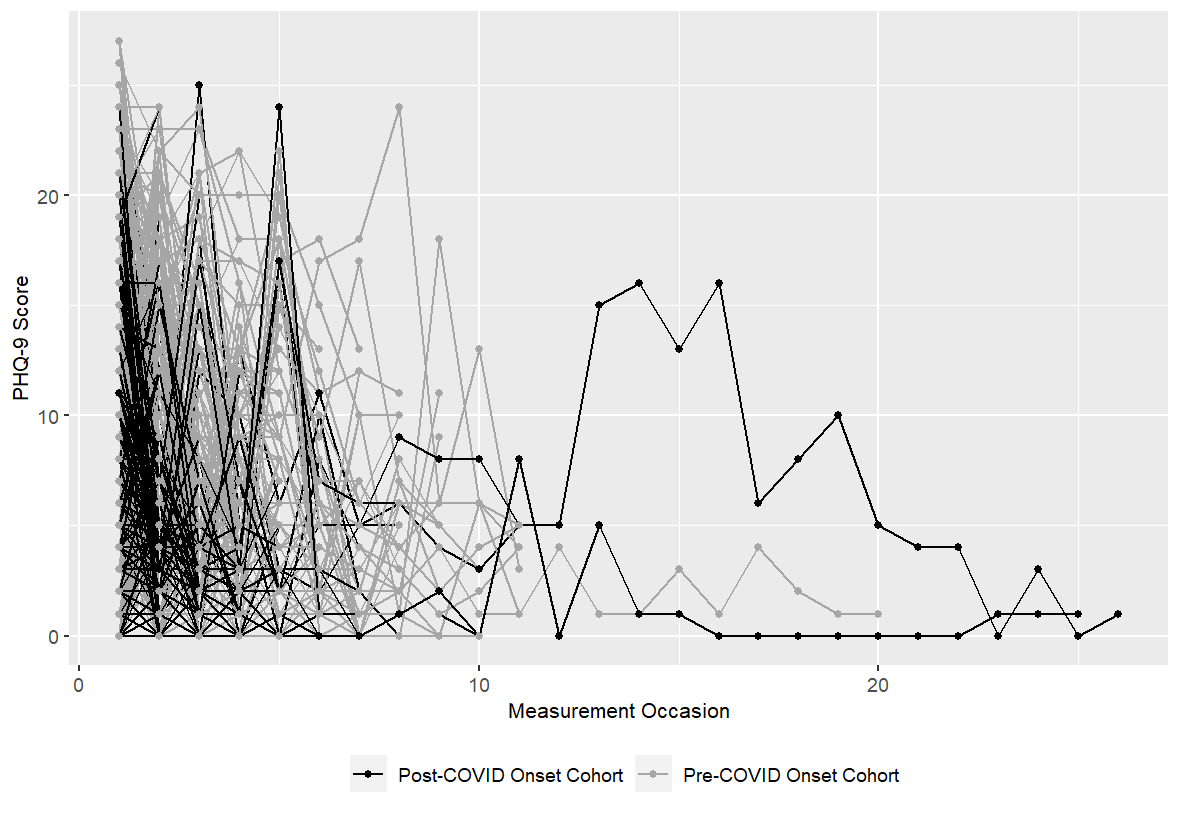

Supplement: Supplementary file 1 [file Data_Sheet_1.docx]
